# Differentiation of human induced pluripotent stem cells into functional lung alveolar epithelial cells in 3D dynamic culture

**DOI:** 10.3389/fbioe.2023.1173149

**Published:** 2023-06-14

**Authors:** Sarah Alsobaie, Tamador Alsobaie, Amal Alshammary, Sakis Mantalaris

**Affiliations:** ^1^ Department of Clinical Laboratory Science, King Saud University, Riyadh, Saudi Arabia; ^2^ Biological Systems Engineering Laboratory, Department of Chemical Engineering, Imperial College London, London, United Kingdom; ^3^ Wallace H. Coulter Department of Biomedical Engineering, Georgia Institute of Technology, Atlanta, GA, United States

**Keywords:** lung development, 3D culture, IPSCs, bioprocess, dynamic condition

## Abstract

**Introduction:** Understanding lung epithelium cell development from human induced pluripotent stem cells (IPSCs) *in vitro* can lead to an individualized model for lung engineering, therapy, and drug testing.

**Method:** We developed a protocol to produce lung mature type I pneumocytes using encapsulation of human IPSCs in 1.1% (w/v) alginate solution within a rotating wall bioreactor system in only 20 days without using feeder cells. The aim was to reduce exposure to animal products and laborious interventions in the future.

**Results:** The three-dimensional (3D) bioprocess allowed cell derivation into endoderm, and subsequently into type II alveolar epithelial cells within a very short period. Cells successfully expressed surfactant proteins C and B associated with type II alveolar epithelial cells, and the key structure of lamellar bodies and microvilli was shown by transmission electron microscopy. The survival rate was the highest under dynamic conditions, which reveal the possibility of adapting this integration for large-scale cell production of alveolar epithelial cells from human IPSCs.

**Discussion:** We were able to develop a strategy for the culture and differentiation of human IPSCs into alveolar type II cells using an *in vitro* system that mimics the *in vivo* environment. Hydrogel beads would offer a suitable matrix for 3D cultures and that the high-aspect-ratio vessel bioreactor can be used to increase the differentiation of human IPSCs relative to the results obtained with traditional monolayer cultures.

## 1 Introduction

An estimated 60 million people worldwide live with lung diseases, such as asthma, emphysema, and chronic obstructive pulmonary disease. Other lung diseases, including pulmonary fibrosis, pneumonia, and lung cancer cause four million deaths every year (Ghaedi et al., 2015). At present, the last resort treatment for end-stage lung disease is lung transplantation, a procedure fraught with problems, including shortage of donors, the risk of graft rejection, and the need for life-long administration of immunosuppressive drugs. Recent studies have suggested that using the patient’s own cells to produce an engineered lung that could be implanted into the patient or used as a bioartificial external support represents a very promising alternative to transplantation (Ott et al., 2010). However, lung tissue engineering capacity has been compromised by the limited availability of human lung epithelial cells for isolation and expansion *in vitro* (Wagner and Griffith, 2010). Lung epithelial cells differentiated from human induced pluripotent stem cells (IPSCs) represent a patient-specific potential source of cells, with the expected advantages of minimal graft rejection issues, abrogating the need for immunosuppressive drugs post-transplantation (Ghaedi et al., 2015).

The lung has a multicellular three-dimensional (3D) architecture including major epithelial cell differences along the proximodistal axis. The most distal region of the lung is comprised by two primary epithelial cell types: type I and type II pneumocytes. The majority of the alveolar surface area (up to 95%) is composed of type I pneumocytes, which are primarily responsible for gas-exchange, while type II pneumocytes are cuboidal cells that secrete alveolar surfactants, such as protein C (Rawlins and Hogan, 2006). Deriving lung epithelia from embryonic stem cells *in vitro* is a complex process that remains unclear (Siti-Ismail et al., 2012). As mentioned above, an alternative strategy would be to use human IPSCs, which can be obtained by genetically reprogramming adult cells (Takahashi and Yamanaka, 2006). These cells can further differentiate into different lineages, including lung epithelium (Siti-Ismail et al., 2012; *Ghaedi* et al., 2014). Reprogrammed cells reset their epigenetic fate map, allowing extracellular cues, such as cell culture conditions, to direct phenotypic changes in these cells. Despite its technical feasibility, generating lung progenitors from human IPSCs has yet to be performed on an appreciable scale capable of satisfying the need for large numbers of fully differentiated cells. This can be accomplished by transitioning from traditional two-dimensional (2D) culture methods to methods supporting 3D geometries, including scaffolds and bioreactors (Tsuchiya et al., 2014). According to recent studies, utilizing 3D like spheroids prove to provide multiple benefits when studying lung development, disease modeling and screening for new drugs than monolayer culture (de Carvalho et al., 2019; Leibel et al., 2020; Bluhmki et al., 2021; Oglesby et al., 2021). 3D culture requires a matrix suitable for culturing anchorage-dependent cells, whose size and structural properties allow efficient gas and nutrient supply as well as waste product removal from all cells. To enable culture of anchorage-dependent cells in 3D conditions, provision of a suitable matrix is necessary. Amongst the different matrices used for this purpose, alginate has been proven to be a suitable material. Semi-permeability of alginate allows diffusion of oxygen and nutrients to the cells and elimination of waste products after encapsulation of cells in this hydrogel (Hwang et al., 2009). We have previously described an integrated, single-step culture technique that resulted in enhanced differentiation of murine ES cells into alveolar epithelial cells using encapsulation and a HARV bioreactor. Selection of the appropriate composition of the alginate hydrogel enabled control of hydrogel degradation ensuring the integrity of the hydrogel. Two-dimensional (2D) and three-dimensional (3D) static cultures are mass transport limited resulting in reduced cell growth, especially within the 3D constructs, since transport is diffusion dependent; ultimately, the metabolic requirements of the tissue formed cannot be supported for long culture periods. To overcome such limitations, we have designed a bioprocess (Siti-Ismail et al., 2012) that involves encapsulation in hydrogels and the use of a rotary cell culture microgravity bioreactor (High Aspect Ratio Vessel; HARV), which produces laminar flow minimizing the mechanical stresses on cell aggregates while providing adequate mass transport and oxygenation and supporting superior 3D tissue-like growth compared to other dynamic culture systems. Semi-permeable alginate beads have been successfully used for this application (Miranda et al., 2010). Alginate has been extensively used for tissue engineering and regenerative medicine purposes and has received FDA approval (E. Klontzas et al., 2020). Its ability to form hydrogels under mild gelation conditions in the presence of ions such as Ca^2+^, Ba^2+^, Sr^2+^ renders it suitable for cell-based applications where exposure to harsh crosslinking buffers can lead to cell damage. When alginate is exposed to a crosslinking solution, L-guluronic residues of adjacent polysaccharide strands are connected forming a hydrogel. Alginate hydrogels possess the advantages of natural biomaterials such as excellent biocompatibility and abundance in nature with a low cost, properties which render it an excellent candidate for cell based regenerative medicine applications (Lee and Mooney, 2012). Generating a lung epithelium may also benefit from a state simulating microgravity, whereby cells are not exposed to turbulence and high shear stress, improving cell-to-cell interactions. Nonetheless, agitation with beads, required to enhance gas and nutrient diffusion, can still be present (Hammond and Hammond, 2001; Klaus, 2001; Nickerson et al., 2004; Purevdorj-Gage et al., 2006). Therefore, the aim of this study is to develop a strategy for the culture and differentiation of human IPSCs into alveolar type II cells using an *in vitro* system that mimics the *in vivo* environment.

## 2 Methods

### 2.1 Cell culture

The human IPSC line IMR90-1 was purchased from WiCell Research Institute Inc. (Madison, WI, UnitedStates). IMR90-1 cells were cultured in Matrigel™-coated six-well plates with 2 mL of complete mTeSR™1 medium per well at 37°C and 5% CO_2_ in a humidified incubator.

### 2.2 3D cultures

3D cultures were established as previously described (Ismail, 2009; Siti-Ismail et al., 2012). Two configurations of 3D cultures were compared in this study, namely, T-flasks and a high-aspect-ratio vessel (HARV) bioreactor, both using alginate hydrogels. Due to the extensive formation of extracellular matrix in the 3D hydrogel cultures, extraction of cells for analysis is limited and was not performed.

### 2.3 Encapsulation technique

The alginate hydrogels were prepared from a phosphate buffered saline (PBS) solution containing low viscosity alginic acid of molecular weight 120,000–190,000 g/mol (1.1% w/v) and bovine gelatin (0.1% v/v), (Sigma-Aldrich, St. Louis, MO, UnitedStates) at pH 7.4. The encapsulation procedure has been previously described (Hwang et al., 2009).

### 2.4 HARV bioreactor

For low-shear, non-turbulent environments, HARVs (Synthecon Inc., Houston, TX, UnitedStates) were used (Botchwey et al*.*, 2001). Each HARV unit had a large radius-to-depth ratio (40 × 10 mm) to provide a substantial surface area on the rear face for gas exchange through a gas-permeable membrane. The average bioreactor volume was 50 mL. The culture vessel was a disposable, sterile, clear plastic cylinder with two sampling/injection Luer lock ports and a half-inch drain/fill port. The media was exchanged every 48 h through the fill port, and air bubbles were removed daily through the Luer valves. The HARV units were attached to a rotator base and incubated as described above for 2D cultures.

### 2.5 Directing the differentiation of human IPSCs into alveolar type II pneumocytes

#### 2.5.1 Induction of endoderm

**FIGURE 1:**
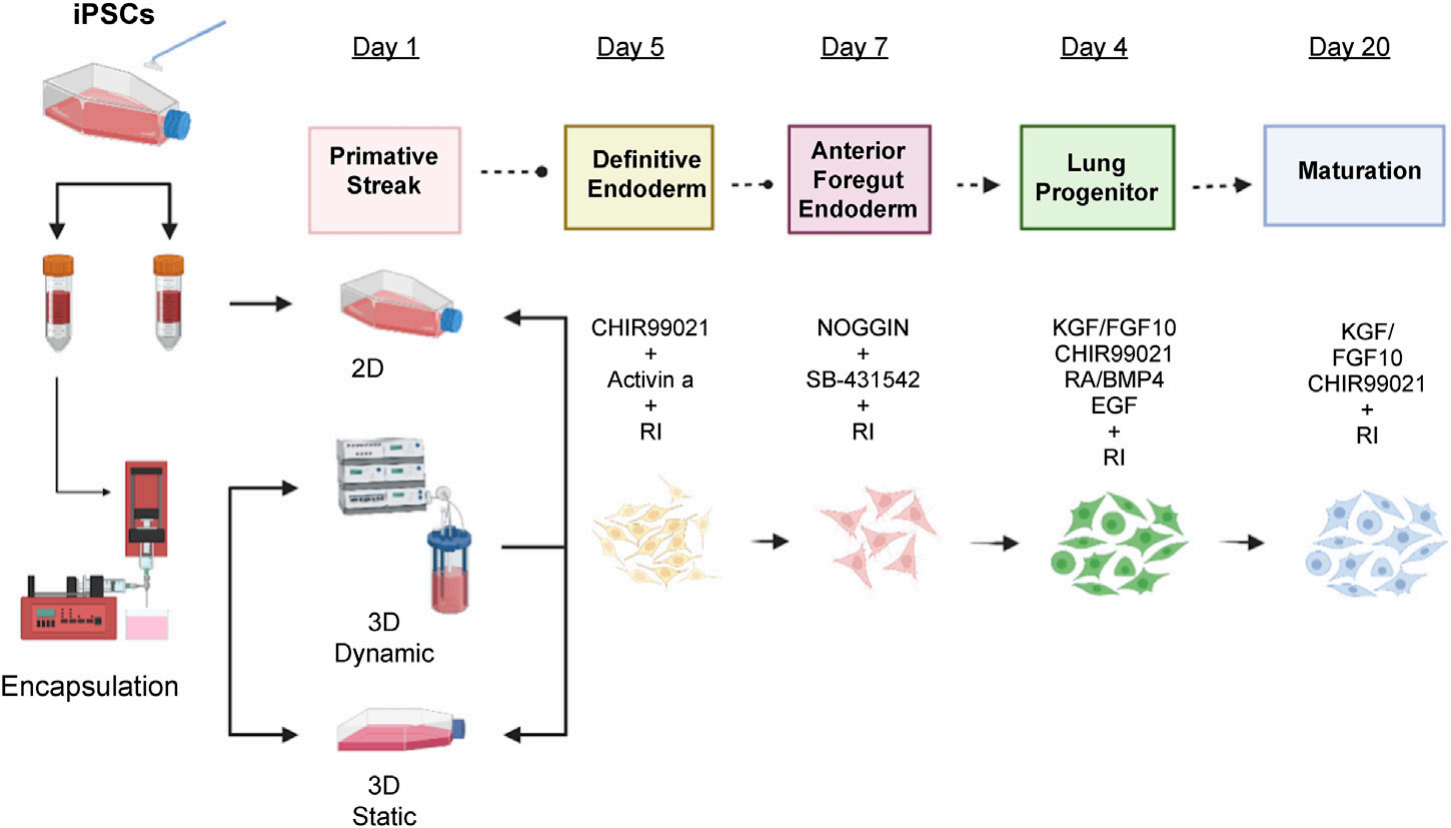
Differentiation protocol for the induction of lung epithelium from human IPSCs. Human IPSCs were encapsulated and cultured in static mode for 24 h in MTeSR™1 media with 10 µM Y-27632. After 24 h, primitive streak formation was induced for 1 day in basal differentiation media composed of DMEM/F12 media supplemented with N2 supplement, B27 supplement, ascorbic acid (50 μg/mL), Glutamax (2 mM), monothioglycerol (0.4 µM), 2% of FBS, and 10 µM Y-27632. Additionally, 1 ng/mL BMP-4 was added to the basal medium. On days two to five, endoderm was induced by adding 3 µM CHIR99021 and human activin A to a final concentration of 100 ng/mL. On days five to seven, 200 ng/mL human Noggin and 10 µM SB-431542 were added to the basal media for anterior foregut endoderm induction and, from days seven to 15, lung progenitor formation was induced with 3 µM CHIR99021, 10 ng/mL human FGF10, 10 ng/mL human FGF7, 20 ng/mL murine EGF, and 500 µM all-trans retinoic acid. Finally, for the maturation stage (days 15–20) 3 µM CHIR99021, 10 ng/mL human FGF10, 10 ng/mL human FGF7, 50 nM dexamethasone, 0.1 mM 8-bromo-cAMP, and 0.1 mM IB were added. DMEM, Dulbecco’s Modified Eagle Medium; FBS, fetal bovine serum; BMP-4, bone morphogenic protein 4; FGF, fibroblast growth factor; EGF, epidermal growth factor.

Human IPSCs were dissociated into single cells, seeded in 2D or encapsulated in 3D cultures and incubated at 37°C overnight with mTeSR™1 medium supplemented with 10 µM Y-27632 (Sigma-Aldrich, Burlington, MA, United State). The next day, 500 alginate beads each were placed in a 50 mL HARV bioreactor (Synthecon Inc. Of Houston, TX), and a T75 flask and resuspended in basal low serum differentiation media (Dulbecco’s Modified Eagle Medium [DMEM]/F12, Life Technologies, Waltham, MA, UnitedStates) supplemented with N2 (Life Technologies), B27 (Gibco, Waltham, MA, UnitedStates), ascorbic acid (50 μg/mL, Sigma-Aldrich), Glutamax (2 mM, Life Technologies), monothioglycerol (0.4 μM, Sigma-Aldrich), 2% fetal bovine serum (FBS) (Life Technologies), and 1% penicillin-streptomycin (Thermo Fisher Scientific, Waltham, MA, UnitedStates). For induction of primitive streak, 10 µM Y-27632, and 3 ng/mL human bone morphogenic protein (BMP4) were added to the basal medium for 24 h. The cells were then resuspended in endoderm induction medium with 10 µM Y-27632, 100 ng/mL human activin A (Tocris Bioscience, Bristol, United Kingdom), and 1 µM CHIR99021 (Tocris Bioscience) for 96 h. Cells were fed every 48 h by replacing half the medium with fresh medium (Figure 1).

#### 2.5.2 Induction of anterior foregut endoderm (AFE)

From days 4–6, the medium was changed to basal medium supplemented with 100 ng/mL of human recombinant Noggin and 10 µM of SB-431542 (Tocris BioScience) (Green et al., 2011).

#### 2.5.3 Induction of lung progenitors

To induce lung progenitors, AFE cells were treated from day 8 to day 17 with a ‘ventralization’ cocktail containing 3 µM CHIR99021 (WNT signaling agonist), 10 ng/mL human fibroblast growth factor (FGF) 10, 10 ng/mL human FGF7, 10 ng/mL human BMP4, 20 ng/mL murine epidermal growth factor (EGF) (optional), and 1 µM all-trans retinoic acid (ATRA) in basal medium. All supplements were purchased from Tocris Bioscience, except for ATRA, which was obtained from Sigma-Aldrich.

#### 2.5.4 Induction of lung epithelial maturation

From day 18/19 to day 25, lung progenitor cells were exposed to basal medium including maturation components, namely, 50 nM dexamethasone (Sigma-Aldrich), 0.1 mM 8-Bromo-cAMP (Sigma-Aldrich), and 0.1 mM 3,7-dihydro-1-methyl-3-(2-methylpro (pyl)-1H-purine-2,6-dione (Sigma-Aldrich). Furthermore, 3 µM CHIR99021, 10 ng/mL human FGF10, and 10 ng/mL human FGF7 were added.

#### 2.5.5 Transmission electron microscopy

Ultrathin sections were prepared as previously described (Ismail, 2009; Siti-Ismail et al., 2012) and observed under a transmission electron microscope (Philips CM10, Amsterdam, Netherlands).

### 2.6 Histology

#### 2.6.1 Paraffin embedding

For histological analysis, alginate beads were fixed in 4% paraformaldehyde for 30 min at room temperature (25°C) and then placed in PBS for 15 min. Fixed beads were dehydrated using a series of increasing ethanol concentrations (50%, 70%, 95%, and 100% [v/v]); 15 min in each ethanol solution. The ethanol was then replaced with xylene by incubating for 15 min three times and subsequently replaced with paraffin-saturated xylene at room temperature overnight. The beads were placed in an oven (60°C) for 20°min, and xylene was completely replaced by liquid paraffin. The samples were embedded and sectioned at 4 µm using a rotary microtome (Thermo Fisher Scientific) and mounted on slides (10149870, Thermo Fisher Scientific). Samples were dried at 37°C overnight prior to staining (Ismail, 2009; Siti-Ismail et al., 2012).

#### 2.6.2 Hematoxylin and eosin staining

The sections were deparaffinized in xylene, rehydrated in decreasing ethanol concentrations and water (Ismail, 2009; Siti-Ismail et al., 2012). Sections were then placed in hematoxylin (Harris solution; VWR) for 30 s and washed in running tap water for 2–3 min. Excess stain was removed by decoloring in 0.5%–1% (v/v) HCl (Sigma-Aldrich) in 70% (v/v) ethanol (Thermo Fisher Scientific) for 2 seconds and then washing with tap water for 5 min. Sections were finally placed in 1% (v/v) eosin (VWR) for 2 min and washed briefly in tap water. Stained sections were dehydrated by submersion in 70% ethanol once and in 100% twice. Finally, sections were cleared in xylene (VWR) and mounted in dibutyl phthalate polystyrene xylene mounting solution (VWR).

### 2.7 Immunohistochemistry

Antigen retrieval for paraffin sections was performed by heating them in citrate buffer (pH 6) for 10 min on full power in a microwave dihydrate buffer (10 mM, pH 6.0) (Sigma-Aldrich) and allowing them to cool and dry. The sections were then washed thrice with PBS (Invitrogen, Renfrew, United Kingdom), followed by permeabilization with 0.1% (v/v) Triton X-100 (Sigma-Aldrich). After washing twice with PBS, non-specific binding was blocked by incubation with 10% (v/v) rabbit, goat, or mouse serum (Vector Laboratories, Burlingame, CA, UnitedStates). The primary antibodies were diluted in PBS with 0.1% (w/v) bovine serum albumin (Sigma-Aldrich) and 0.01% (w/v) sodium azide (Sigma-Aldrich). Sections were incubated with primary antibodies overnight at 4°C (Table 1). The sections were then washed thrice with PBS and incubated for 1 h with biotinylated goat anti-rabbit secondary antibody diluted 1:250 in PBS (Vector Laboratories). An ABC kit was applied for 30 min and a 3,3′-diaminobenzidine kit for 2–10 min to stain the nuclei (Vector Laboratories).

**TABLE 1:** List of primary antibodies for immunocytochemistry staining.

*Antigen*	*Primary Antibody*	*Catalogue no.; Manufacturer*	*Dilution*
Pro-SFTPC	Rabbit polyclonal antibody	AB3786; Millpore	1:1,000–1:2000
Pro-SFTPB	Rabbit polyclonal antibody	AB3430; Millpore	1:4000–1:8000
GAPDH	Goat polyclonal IgG	sc-9890; Santa Cruz	1:50–1:500
FOXA2	Goat polyclonal IgG	sc-6554; Santa Cruz	1:50–1:500
TTF	Rabbit monoclonal IgG	AB76013; Abcam	1:100–1:1,000

Abbreviations: pro-SFTPC, surfactant protein C; pro-SFTPB, surfactant protein B; GADPH, glyceraldehyde-3-phosphate dehydrogenase; FOXA2, forehead box A2; TTF, thyroid transcription factor.

### 2.8 RNA extraction

RNA was extracted using a total RNA isolation kit (Qiagen, Hilden, Germany) according to the manufacturer’s protocol. RNA concentrations were measured using a spectrophotometer (ND-1000, NanoDrop Technologies, Wilmington, DE, UnitedStates). Samples with a 260/280 ratio of ∼2.0 were used for further processing.

### 2.9 cDNA synthesis

To prepare RNA for cDNA synthesis, 1–2 µg of RNA was diluted in diethyl pyrocarbonate-treated water (Thermo Fisher Scientific) and denatured for 5 min at 65°C to remove the secondary structure. The 20 µL reaction mixture also contained reverse transcriptase, (M-MLV RT, Promega, Madison, WI, UnitedStates), 4 mM dNTPs (Promega), 2.5 µM Oligo dT16 primers (Applied Biosystems, Waltham, MA, UnitedStates) and was incubated for 1 h at 37°C. Reverse transcription was terminated by incubation at 95°C for 5 min.

For PCR amplification, sense and anti-sense primers were designed using the Primer Express 2 software (Applied Biosystems). Oligonucleotides were purchased from Invitrogen (Table 2).

**TABLE 2:** List of qRT-PCR primer sequences.

*Gene*	*Forward primer (5′−3′)*	*Reverse primer (5′−3′)*
*GAPDH*	GAC​AAC​AGC​CTC​AAG​ATC​ATC​AG	ATG​GCA​TGG​ACT​GTG​GTC​ATG​AG
*NKX2.1*	TCG​CTC​CAG​CTC​GTA​CAC​C	GGA​CGT​GAG​CAA​GAA​CAT​G
*CXCR4*	CAC​CGC​ATC​TGG​AGA​ACC​A	GCC​CAT​TTC​CTC​GGT​GTA​GTT
*FOXA2*	GGGAGCGGTGAAGATGGA	TCA​TGT​TGC​TCA​CGG​AGG​AGT​A
*AQ5a*	ACT​GGG​TTT​TCT​GGG​TAG​GG	ATG​GTC​TTC​TTC​CGC​TCT​TC
*®-actin*	CAT​GTA​CGT​TGC​TAT​CCA​GGC	CTC​CTT​AAT​GTC​ACG​CAC​GAT
*CFTR*	CTA​TGA​CCC​GGA​TAA​CAA​GGA​GG	CAA​AAA​TGG​CTG​GGT​GTA​GGA
*SFTPC*	CAC​CTG​AAA​CGC​CTT​CTT​ATC​G	TGG​CTC​ATG​TGG​AGA​CCC​AT
*LAMA5*	CCT​CGT​CCT​CCA​ATG​ACA​C	GCGCTGCAGTCACAATTC
*FN1*	AGG​AAG​CCG​AGG​TTT​TAA​CTG	AGG​ACG​CTC​ATA​AGT​GTC​ACC
*OCT3/4*	CCT​CAC​TTC​ACT​GCA​CTG​TA	CAG​GTT​TTC​TTT​CCC​TAG​CT
*PAX6*	TGT​CCA​ACG​GAT​GTG​TGA​T	TTT​CCC​AAG​CAA​AGA​TGG​AC

Abbreviations: SFTPC, surfactant protein C; GADPH, glyceraldehyde-3-phosphate dehydrogenase; FOXA2, forehead box A2; CFTR, cystic fibrosis transmembrane conductance regulator; CXCR4, C-X-C chemokine receptor 4; LAMA5, laminin 5; FN1, fibronectin; PAX6, paired box protein 6; qRT-PCR, quantitative reverse transcription polymerase chain reaction.

### 2.10 Polymerase chain reaction (PCR)

Quantitative real time PCR analysis of cDNA was performed using the StepOne Real-Time PCR System (Applied Biosystems) and the SYBR green detection system (SensiFAST™ SYBR^®^ Hi-ROX Kit, Thermo Fisher Scientific). Reactions were performed in 96-well plates. The thermocycler settings were as follows: 95°C for 2 min, 40 cycles of 95°C for 5 s, and 60°C for 30 s. The reaction was completed with a dissociation step: 15 s at 95°C, 15 s at 60°C, and 15 s at 95°C. A single peak in the resultant dissociation plot represents a single specific product. Each sample was analyzed in triplicate, and negative control reactions without cDNA were included in each experiment. Glyceraldehyde-3-phosphate dehydrogenase and ®-actin were used for normalization.

### 2.11 Cell viability assays

Cell viability was assessed using the CellTiter-Glo Luminescent Cell Viability Assay (Promega) according to the manufacturer’s instructions. Briefly, a single reagent was added for cell lysis and generation of a luminescent signal proportional to the amount of ATP present in the cells, which is directly proportional to the number of viable cells.

### 2.12 Live/dead assay

*In situ* live and dead cells were visualized using the LIVE/DEAD^®^ Viability/Cytotoxicity Assay Kit (Invitrogen). This kit provides a two-color fluorescence cell viability assay allowing simultaneous detection of live and dead cells using two probes that measure two recognized parameters of cell viability, intracellular esterase activity and plasma membrane integrity. Live cells are distinguished by the presence of ubiquitous intracellular esterase activity, determined by the enzymatic conversion of the virtually non-fluorescent, cell-permeant calcein AM to the intensely fluorescent calcein. The polyanionic dye calcein is retained within live cells, producing intense uniform green fluorescence (excitation [EX]/emission [EM] ∼495 nm/∼515 nm). Ethidium homodimer-*1* (EthD-1) enters cells with damaged membranes and undergoes a 40-fold enhancement of fluorescence upon binding to nucleic acids, thereby producing bright red fluorescence in dead cells (EX/EM ∼495 nm/∼635 nm). EthD-1 is excluded by the intact plasma membrane of live cells. The determination of cell viability depends on these physical and biochemical properties of cells. A 4 mM EthD-1 and 2 mM calcein AM solution (Invitrogen) diluted in 10 mL PBS (Life Technologies) was added to live cells or alginate beads and incubated at room temperature for 30 min in the dark. After incubation, the solution was aspirated, and a small amount of PBS was added to the cells to prevent dehydration. The cells and beads were then photographed within 30 min using an IX70 inverted fluorescent microscope (Olympus, Tokyo, Japan) to assess the number of live and dead cells.

### 2.13 MTS assay

The MTS substrate (CellTiter 96^®^ AQ_ueous_ One Solution Cell Proliferation Assay (MTS) kit, Promega) was prepared in a cell culture medium, added to cells in culture at a final concentration of 0.2–0.5 mg/mL of culture medium, and incubated for 1–4 h. MTS standard curve was calculated using known cell number.

### 2.14 Western blotting

Beads were washed twice with cold PBS and resuspended in 1 mL radio-immunoprecipitation Assay (RIPA) buffer with protease inhibitors (Thermo Fisher Scientific). Beads were briefly vortexed and incubated on ice for 10 min. The extracts were then centrifuged at 1,000 *g* at 4°C for 15 min, and the supernatants were collected as the protein lysates. Chemiluminescent substrate was used (Pierce, Thermo Fisher, Scientific) according to the manufacturer’s instructions (Table 3). Membranes were developed and densitometric analysis of the films was performed using the Quantity One 1-D Analysis Software, 2003 version (BioRad) (30 min exposure).

**TABLE 3:** List of primary antibodies for immunoprecipitation and Western blotting.

*Antigen*	*Primary Antibody*	*Catalogue no.; Manufacturer*	*Dilution*
C (pro-SFTPC)	Polyclonal antibody Rabbit	AB3786; Millpore	1:1,000–1:2000
B (pro-SFTPB)	Polyclonal antibody Rabbit	AB3430; Millpore	1:4000–1:8000
GADPH	goat polyclonal IgG	sc-9890; Santa Cruz	1:50–1:500

Abbreviations: pro-SFTPC, surfactant protein C; pro-SFTPB, surfactant protein B; GADPH, glyceraldehyde-3-phosphate dehydrogenase.

### 2.15 Statistics

Data are presented as the mean ± standard error of the mean. Comparisons between two treatment groups were performed using an unpaired two-tailed Student’s t-test. Comparisons between multiple groups were performed by one-way analysis of variance using Dunnett’s *post hoc* test with the GraphPad software package (GraphPad Software Inc., San Diego, CA, UnitedStates), and *p* < 0.05 was considered statistically significant. For all experiments, a minimum of three biological replicates were performed.

## 3 Results

### 3.1 Cell proliferation and viability in static and dynamic 3D cultures

**FIGURE 2:**
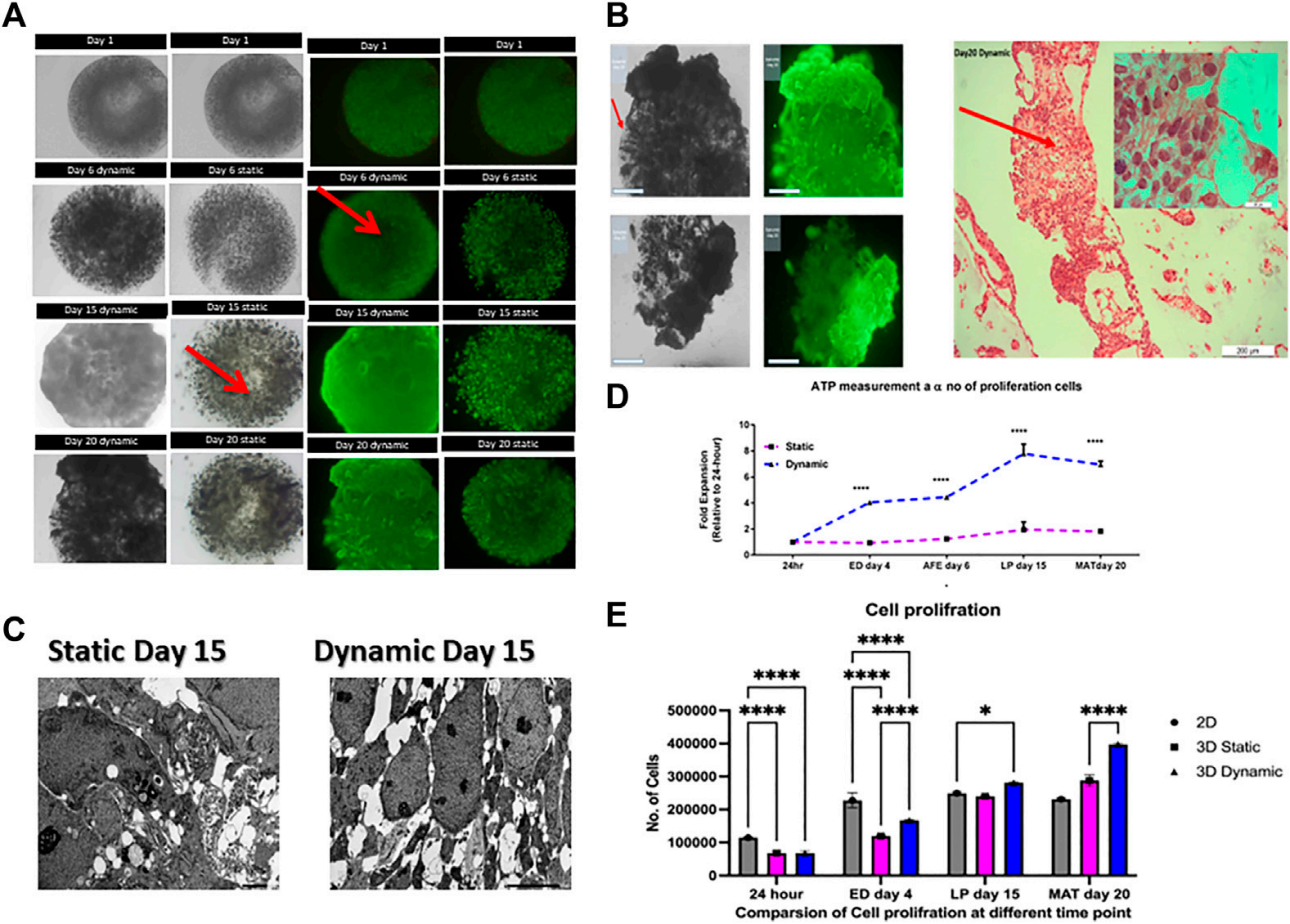
Comparison of cell proliferation in static and dynamic 3D cultures. **(A)** Images of live/dead staining show increased cell growth throughout the differentiation process. Red arrows show the necrotic cores that occur under static conditions. Scale bar, 2 mm; **(B)** The image indicates that there was a high degree of movement, migration of initially immobilized cells, and formation of tissue-like structures in 3D dynamic culture by day 20. Representative images of hematoxylin and eosin-stained cells, their extracellular matrix formed tissue-like assemblies and the cells proliferated more in 3D dynamic conditions by day 20. Scale bars for 2 mm and 200 μm; **(C)** TEM of 3D cultures at day 15 revealed the presence of necrotic cells or apoptotic bodies (red arrows) and a heterogeneous cell population in static condition whereas no necrotic or apoptotic structures were found in the homogenous dynamically cultured cells. Scale bar, 5.0 µm. 3D, three-dimensional; IPSC, induced pluripotent stem cells; TEM, transmission electron microscopy. **(D)** Cell Titer Glo profile of encapsulated human IPSCs. Proliferation (normalized to day one, error bars show one standard deviation, n = 3 biological replicates. **** = *p* < 0.0001; **(E)** Proliferation plot based on the results of MTS assay assay. Cellular proliferation measured at different time point. All data represt the mean of there independent experiments. P-valueobtained by two way analysis of variance higher on ****p ≤ 0.0001, ***p ≤ 0.001, and **p ≤ 0.01.

Cell viability was evaluated by live/dead staining with calcein AM and ethidium bromide, and cells were observed under a fluorescence microscope. Cell viability assays on days 1, 6, 15, and 20 (Figure 2A) showed that viability was high in dynamic 3D cultures, with no evidence of a necrotic core (Figure 2A). Furthermore, an “O-ring”-like growth pattern was observed in 3D static conditions, possibly due to nutrient limitation in the center (Figure 2A) as illustrated with red arrow.

On day 20, large tissue-like cell aggregates had formed around the beads (Figure 2A). In contrast to the static cultures, cells aggregated on the bead periphery, allowing interactions with neighboring cells and the extracellular matrix (ECM), as would occur in normal tissue. As shown in Figure 2B, the cells remained attached but grow outside the beads forming tissue-like structure as illustrated with hematoxylin and eosin staining. One possibility is that the constant movement and shear forces in dynamic culture may physically disrupted the alginate beads or increased availability of nutrient and oxygen, which may have fueled the proliferation of the cells and put additional stress on the alginate matrix. This result is in agreement with previously published observations, which tentatively support the ability of the hydrogel to be degraded, and indicate the permeability of its internal architecture (Zehnder et al., 2015). Taken together, these results indicate that the vessel rotation is sufficient for gas and metabolic exchange.

In addition, transmission electron microscopy on day 15 showed the presence of necrotic and apoptotic bodies in static cultures but not in dynamic cultures (Figure 2C). Transmission electron microscopy showed necrotic cells and apoptotic bodies in samples from cells grown under static conditions but not for those from dynamic conditions. Additionally, the live/dead assay showed increased cell density on the bead edge under static conditions and a reduction in the center of the beads. This is possibly due to oxygen transport limitations within the microcapsules, which might have led to cellular necrosis in the core (Huang et al., 2012). The difference between dynamic and static conditions was analyzed using a Cell Titer Glo 3D assay, which confirmed that viability was higher in dynamic cultures than in static cultures at all time points (*p* < 0.0001, Figure 2D). Cell proliferation was measured using MTS assay showing the yield rate about 45,000 and 30000/beads on dynamic and static condition respectively and 20,000 cells per 96 well plate for 2D at day 20 (Figure 2F). Live/dead imaging and ATP measurements using a Cell Titer Glo 3D assay showed that both static and dynamic 3D cultures enhanced cell proliferation. Cell proliferation in 3D dynamic cultures resulted in an eight-fold higher yield of cells than that in static cultures; this difference was statistically significant (*p* < 0.0001).

### 3.2 Induction of human IPSC differentiation into type II alveolar cells

**FIGURE 3:**
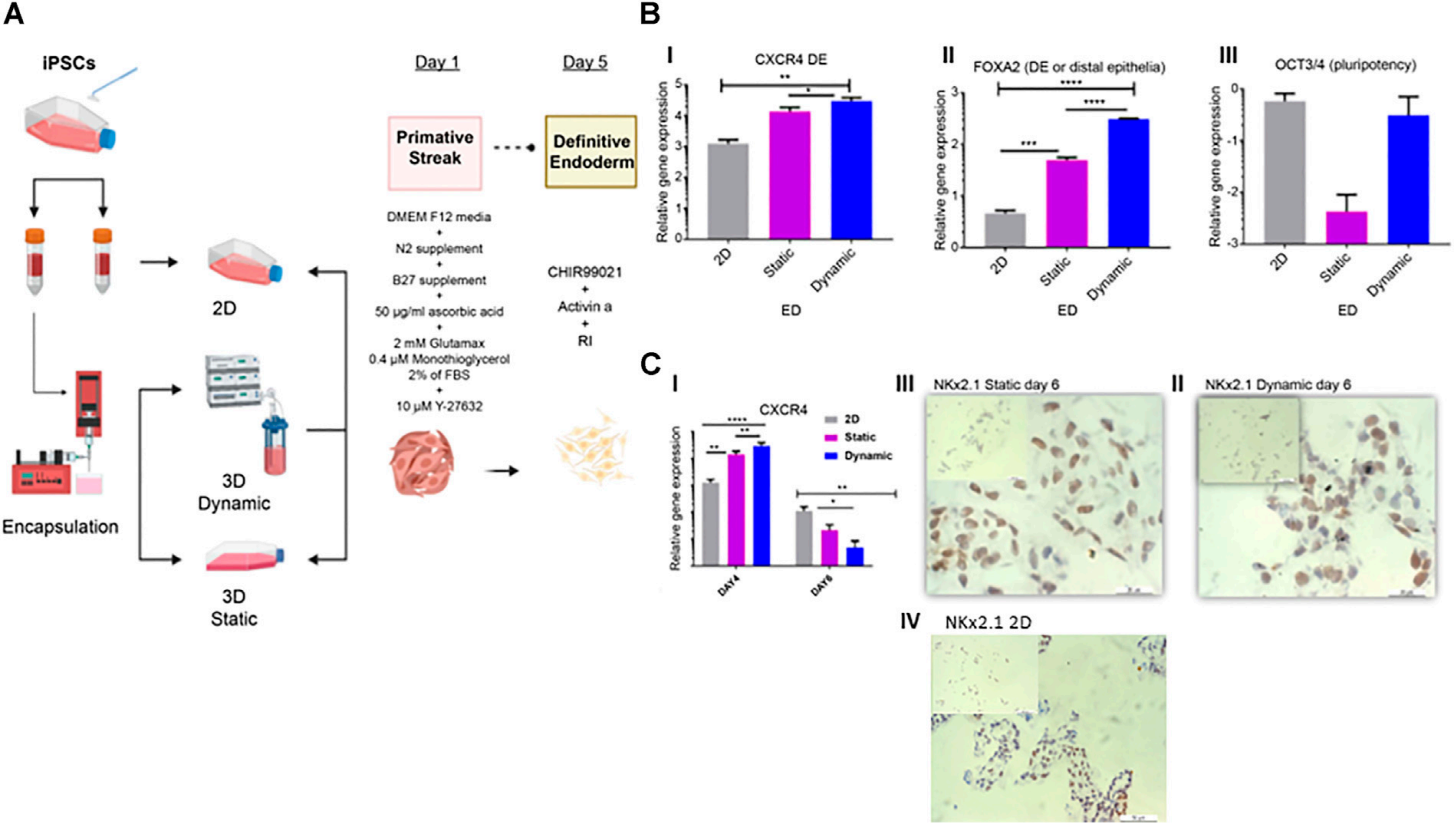
Characterization of endoderm induction (ED). **(A)** Differentiation protocol for the induction of lung epithelium from human IPSCs. After 24 h in static cultures, cells were differentiated in four stages spanning 20 days of culture. **(B)** On day four, qRT-PCR of endoderm-associated markers CXCR4, FOXA2, and pluripotency marker OCT3/4 was performed. **(C)** Gene expression and immunohistochemistry analyses conducted on day 6 and representative immunohistochemistry images showing Nkx2.1, on day 6 in static (II), dynamic (III), and 2D culture (IV) cultures. Scale bar, 20 µm. Immunohistochemistry controls are presented in Supplementary Figures S1, 2. Error bars show the mean fold change on day 4 and day 6, error bars show one standard deviation, *n* = 3 biological replicates. Gene expression was normalized to day 0 (2^−ΔΔCT^ method). *p*-values were obtained using one-way ANOVA, where ****p ≤ 0.0001, ***p ≤ 0.001, and **p ≤ 0.01. IPSC, induced pluripotent stem cells; qRT-PCR, quantitative reverse transcription polymerase chain reaction; CXCR4, C-X-C chemokine receptor 4; FOXA2, forehead box A2; RI, rock inhibitor; ANOVA, analysis of variance.

We examined the effect of static and dynamic 3D cultures on the induction of type II alveolar cells (Figure 3). We analyzed the expression of markers associated with type II alveolar cells on days 4, 6, 15, and 20 during the differentiation process. The genetic markers analyzed were: C-X-C chemokine receptor (CXCR)4, an endoderm marker; forkhead box A2 (FOXa2), the endodermal transcription factor and distal epithelia marker; OCT3/4, a pluripotency marker; Nkx homeobox-1 (Nkx2.1), a lung progenitor marker; surfactant proteins (SFTP), as markers for type II alveolar cells; cystic fibrosis transmembrane conductance regulator (CFTR), a marker of proximal epithelia; and AQ5a, a marker for type I cells.

On day 4, both CXCR4 Figures 3B, I) ***p ≤ 0.001 and FOXa2 (Figures 3B, II) ****p ≤ 0.0001, were significantly upregulated in dynamic cultures compared to static and control 2D cultures (Figure 3B, I, II). Expression of the pluripotency marker OCT3/4 did not differ significantly in this time point between control and 3D cultures (Figures 3B, III), as previously described (Kubo et al., 2004; D’Amour et al., 2005; Sturgeon et al., 2014). However, OCT3/4 was considerably downregulated in static cultures.

On day 6, CXCR4 expression was reduced compared to that on day 4 (Figures 3C, I). This reduction was observed in both static and dynamic cultures, with significant differences between 3D cultures and the 2D controls. This suggests that induction of anterior foregut endoderm was more effective in the 3D cultures (Figures 3C, I). On day 6, the first marker for lung progenitors, thyroid transcription factor 1 (TTF-1)/Nkx2.1 was detected by immunohistochemical staining in dynamic, static and 2D cultures (Figure 3C, II–IV).

**FIGURE 4:**
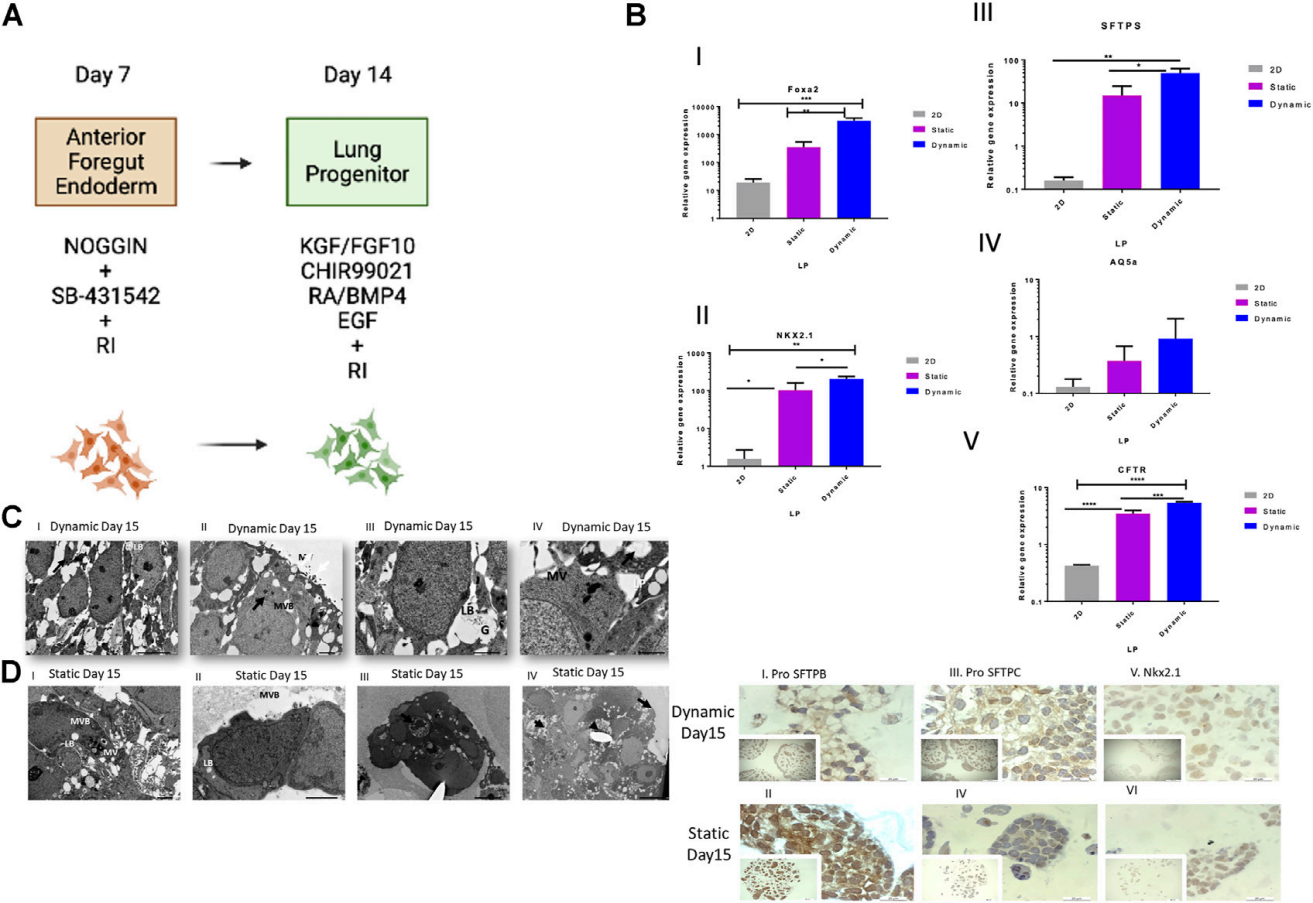
Cell and genetic analysis of progenitor differentiation markers on day 15 between control and two experimental groups (static and dynamic). **(A)** Lung progenitor protocol. **(B)** qRT-PCR analysis of the expression of selected markers on day 15. qRT-PCR of lung progenitor markers. Bars show the mean fold change on day 15; error bars show one standard deviation; *n* = 3 biological replicates; data normalized to day 0 (2^−ΔΔCT^). *p*-values obtained by one-way ANOVA ****p ≤ 0.0001, ***p ≤ 0.001, **p ≤ 0.01 and *p ≤ 0.05. **(C)** TEM of dynamic cultured cells on day 15. (I) Loosely organized membranes of immature lamellar bodies (red arrows). Scale bars, 5 µm. II) MV = surface of the epithelial layer or surface microvilli, MVB = multivesicular bodies, and red arrows show electron-dense lamellar bodies. Scale bar, 1 μm; III) LB = lamellar bodies; G = glycogen particles trapped inside immature lamellar bodies. Scale bar, 2 µm. IV) Lipid droplets (LP) and microvilli (MV) and immature lamellar bodies (red arrow). Scale bars, 2 µm. **(D)** TEM of static cultured cells on day 15. TEM images: (I) MV = microvilli. Scale bars, 5 μm; II) LB = an immature lamellar body, MV = microvilli, small red arrow indicates extracellular space containing small debris, typical of an apoptotic cell. Scale bars, 2 μm; III) LB = an immature lamellar body; IV) Cells formed aggregates inside the beads and showed heterogeneity in cell morphology. The red arrow points to a typical apoptotic cell. Scale bar, 10 μm; long arrow points to the surface of the epithelial layer or the surface of cells (no microvilli). The small arrow points to the extracellular space showing small debris, typical of an apoptotic cell. Scale bars, 10 µm. **(E)** Immunohistochemical analysis of selected markers on day 15. Representative immunohistochemistry images with antibodies against pro-SFTPB (I and II), pro-SFTPC (III and IV) and Nkx2.1 (V and VI) on day 15 in static and dynamic cultures. Scale bar, 20 µm. RI, rock inhibitor; ANOVA, analysis of variance; TEM, transmission electron microscopy; pro-SFTPC, surfactant protein C; pro-SFTPB, surfactant protein **(B)**.

On day 15, further differentiation towards proximal and distal lung bud progenitors (Figure 4A) was confirmed by expression of FOXA2, Nkx2.1, and CFTR. These markers were significantly upregulated in the dynamic cultures, as compared to static and 2D cultures FOXA2 (**p ≤ 0.01 Static, ***P 2D, Nkx2.1 and *p ≤ 0.05 static, **p ≤ 0.01 2D, CFTR, ***p ≤ 0.001 static, ****p ≤ 0.0001 2D) (Figure 4B, I, II, IV), especially FOXA2, the marker for distal epithelium. In addition, we examined the early differentiation of cells expressing surfactant protein-C (SFTPC), because functional alveolar epithelial type II cells (AEC2) are derived from SFTPC^+^ distal lung cells. Expression of SFTPC, a marker for the pathway towards functional AEC2 cells, was also significantly upregulated in the dynamic cultures compared to that in the static and 2D cultures (*p* ≤ 0.05 and *p* ≤ 0.01, respectively) (Figure 4B, III). Interestingly, AQ5a, a marker of type 1 cells, was not significantly upregulated at this time point (Figure 4B, IV).

Transmission electron microscopy on day 15 of culture for static (Figure 4C) and dynamic conditions (Figure 4D) showed loosely organized membranes of immature lamellar bodies in dynamic conditions (Figures 4D, I), microvilli (MV), surface of the epithelial layer or surface MV, multivesicular bodies (Figures 4D, II), lamellar bodies, glycogen particles trapped inside immature lamellar bodies (Figures 4D, III), as well as lipid droplets, MV, and immature lamellar bodies (Figures 4D, IV). The expression of the corresponding proteins in type II cells including Nkx2.1, pro-SFTPC, and pro-SFTPB was confirmed by immunohistochemistry. Both 3D cultures expressed all these protein markers (Figure 4E, I–V). Surprisingly, the results showed protein markers of mature type II cells, contradicting the findings of the study from which this protocol was adapted as Huang et al. showed no mRNA expression of lung and airway markers in day 15 cultures (Huang et al., 2013). Expression of pro-SFTPC at this stage was detected by Western blotting in static and dynamic bead cultures, but not in 2D control cultures, indicating the maturation of type II cells at an early stage of the protocol (Supplementary Figure S3).

**FIGURE 5:**
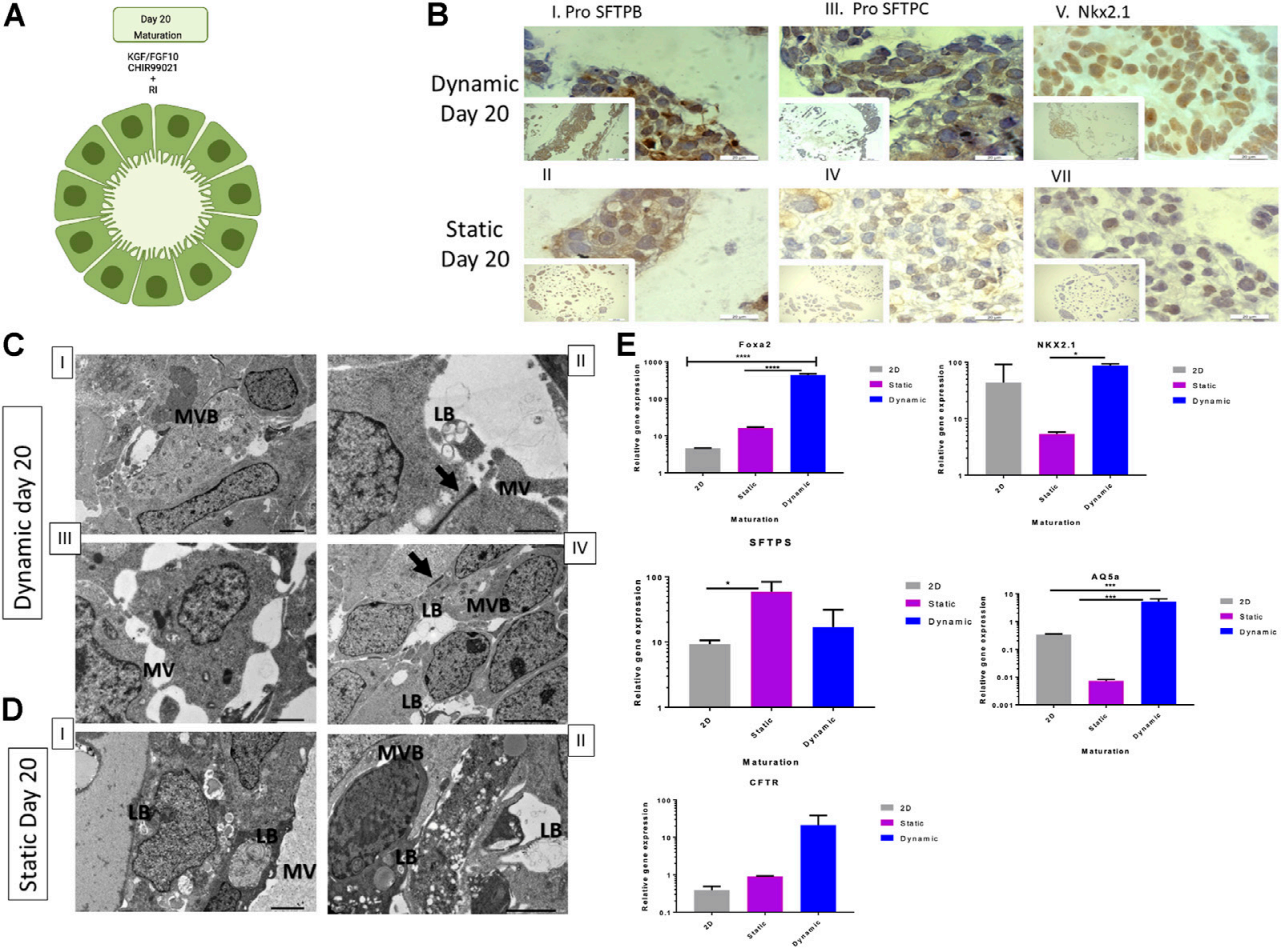
Cell and genetic analysis of lung maturation markers on day 20 between control and two experimental groups (static and dynamic). **(A)** Lung maturation stage induction protocol. **(B)** Representative immunohistochemistry images with antibodies against pro-SFTPB (I and II), pro-SFTPC (III and IV), and Nkx2.1 (V and VI) on day 20 under dynamic and static conditions. Scale bar, 20 µm. RI, rock inhibitor; TEM, transmission electron microscopy; pro-SFTPC, surfactant protein C; pro-SFTPB, surfactant protein B; qRT-PCR, quantitative reverse transcription polymerase chain reaction; ANOVA, analysis of variance. **(C)** Representative TEM microscopy images of dynamic cultured cells on day 20. Cells derived from alveoloi spheres. MVB = multivesicular bodies; red arrow indicates a tight junction; LB = formation of lamellar bodies, MV = microvilli. Scale bars, 6 µm for images I and IV. Scale bars, 2 µm for images II and III. Immunohistochemistry analysis conducted on day 20. **(D)** Representative TEM microscopy images of static cultured cells on day 20. Cells derived from alveoli spheres. Scale bars, 2 µm for images I and II. **(E)** qRT-PCR of lung progenitor markers; mean fold change on day 20 *versus* day zero (2^−ΔΔCT^) ± SD, *n* = 3 biological replicates on day 15; ****p ≤ 0.0001, ***p ≤ 0.001, **p ≤ 0.01, and *P % 0.05 by one-way ANOVA.

Finally, on day 20 (Figure 5A), we evaluated expression levels of the same genes assessed in the previous stages to determine the maturation of type II cells. As shown in Figure 5B, FOXA2 (Figure 5E) expression was significantly higher in 3D dynamic cultures (*p* ≤ 0.0001) when compared to that in other conditions. Nkx2.1 and pro-SFTPC were downregulated in 3D dynamic bead cultures compared to day 15 (Figure 5E), while AQ5a was significantly upregulated (*p* ≤ 0.001) in dynamic cultures (Figure 5E). The gene expression profile of the static culture on day 20 was insufficient to draw a conclusion (Figure 5E). The transport of nutrients and gases may have caused different populations of cells to co-exist within the beads or cells were in varied stages, including quiescent and transit-amplifying cells (Zhang et al*.*, 2015). In 2D cultures, cells expressed markers related to the early lung progenitor stage (Figure 5E). Immunohistochemical staining (Supplementary Figure S3) confirmed the conclusions drawn from the gene expression data.

In summary, gene expression analysis showed preferential upregulation of differentiation markers in dynamic bead cultures in the HARV bioreactor. On days 6, 15, and 20, we performed immunohistochemical staining of the beads in the static and HARV groups. The cells were stained with antibodies against TTF-1 and Nkx2.1 on day 6 to confirm AFE and lung bud specification (Figure 3C, II, III). To test for type II pneumocyte differentiation, beads grown under both conditions were immuno-stained with antibodies against TTF-1/Nkx2.1, pro-SFTPC, and pro-SFTPB. Furthermore, all bead cultures showed immunoreactivities for all markers on days 15 (Figure 4E, I–VII) and 20 (Figure 5B, I–VII), specifically for SFTPB, SFTPC, and NKx2.1. In addition, SFTPC expression was confirmed by Western blotting. Both 3D conditions showed higher expression of SFTPC than 2D conditions (Supplementary Figure S3).

At the maturation stage (day 20), both static and dynamic bead/aggregate cultures showed higher potential to produce type II cells than the control group (2D culture) based on gene expression data. The increase in expression of type I markers and decrease in expression of type II markers highlight the ability of mature type II cells to generate their progenitors (Jacob et al*.*, 2017).

### 3.3 Differentiated lung epithelium cells produced in 3D cultures are functional

Despite the early *in vivo* expression of SFTPC and other surfactant markers in alveolar progenitors during week 12–15 of human gestation (*Khoor* et al., 1994), the maturity of functional lamellar bodies *in vivo* becomes significant after week 24 in type II alveolar pneumocytes (Jacob et al., 2017). Using transmission electron microscopy, we assessed whether cells grown in 3D cultures express the highly specialized organelles of type II cells, such as functional lamellar bodies, microvasculature, and microfilaments.

On day 15, cells cultured in a 3D dynamic environment had loosely organized membranes, glycogen particles trapped inside immature lamellar bodies, surface microvilli, and multivesicular bodies (Figure 4D, I–IV), which are considered phenotypic markers of typical immature lamellar bodies (Ridsdale and Post, 2004). In static cultures, lamellar bodies appeared to be immature, and no MV or multivesicular bodies were detected. Rather, the cells showed some debris and typical apoptotic morphology (Figures 4C, I, II).

On day 20, we detected functional lamellar bodies, the classic marker of AEC2 maturity and other highly specialized organelles, namely, multivesicular bodies, and no MV were detected in 3D dynamic cultures (Figure 5E I, IV) (Castranova et al*.*, 1988). However, on day 20, static cells showed AEC2 structures like multivesicular bodies, corroborating the formation of mature lamellar bodies with MV (Figure 5DI, II).

### 3.4 Extracellular matrix (ECM) production in 3D dynamic culture enhances cell proliferation and differentiation

**FIGURE 6:**
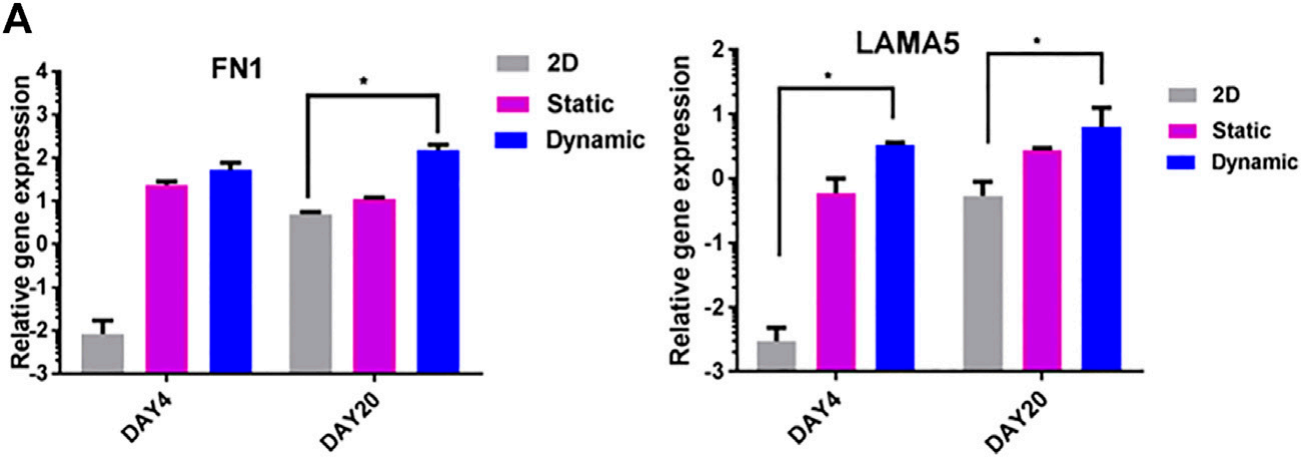
ECM gene expression in static and rotary environments. **(A)** Expression of fibronectin (FN1) and laminin (LAMA5) genes on days 4 and 20. Significant differences between 3D dynamic cultures and control cultures were observed on day 4, and to a lesser extent on day 20 (2D).

The effects of 3D dynamic culture on the expression of fibronectin 1 (FN1) and laminin 5 (LAMA5) were evaluated using quantitative real-time PCR (Figure 6A). In contrast to 2D controls, cells cultured in 3D dynamic conditions showed significantly upregulated expression of FN1 and LAMA5 on day 4, and to a lesser extent, on day 20 (*p* < 0.05) (Figure 6A). This is an important regulator of distal lung epithelial cell differentiation and lung maturation (Nguyen and Senior, 2006).

Numerous reports have suggested that the phenotypic behavior of cells can be influenced by their 3D matrix structure and organization (Salgado et al., 2004). In summary, ECM synthesis in 3D dynamic culture enhanced cellular proliferation and differentiation into type II lung epithelium.

## 4 Discussion

In the present study, we elucidated conditions that promote 3D tissue engineering of type II pneumocytes. Human IPSCs were encapsulated inside alginate hydrogel beads, and dynamic movement was simulated using a HARV bioreactor. In addition, the impact of 3D culture dynamics on the induction of differentiation, proliferation, survival, molecular changes, and functionality of type II pneumocytes was investigated. To the best of our knowledge, this is the first study to demonstrate the direct differentiation of human IPSCs towards lung epithelium using alginate encapsulation and a HARV bioreactor. The expression of SFTPC, SFTPB, and Nkx2.1, specific markers of type II lung epithelial pneumocytes, at the gene and protein levels was detected as early as day 15 of differentiation. Conversely, Huang et al. did not identify the expression of any of these markers at these stages (Huang et al., 2013). The early induction of type II epithelium in our protocol suggests a promotive role for 3D alginate beads in lung induction (Anton, et al., 2015; Jha et al., 2016; Chandrasekaran et al., 2017).

The differentiation conditions used in this study demonstrate the ability of both static and dynamic 3D cultures to deliver mature lung epithelial cells from human IPSCs. However, for large-scale cell production, dynamic culture provides an improved environment for cell growth and proliferation. This difference could perhaps be attributed to the fact that the dynamic culture environment mitigates the stress caused by waste product accumulation and improves cellular oxygenation (Unsworth and Lelkes, 1998; Yuge et al., 2006; Zhang et al., 2015).

Our method provides an optimized approach, combining suitable biomaterials and bioprocess conditions to produce cells to be used in transplants. Cells naturally exist in 3D configuration providing specific biophysical and biomechanical signals according to the type of tissue, which influences cell functions such as differentiation and gene expression (Souza et al., 2010). We envision that implantation of the cellularized 3D hydrogels would be the best approach in potential clinical applications since the hydrogels are biodegradable and to preserve the extensive tissue formation *in situ*. It is well established that alveolar epithelial type II cells respond to mild shear stress and regulate surfactant secretion in response (Mahto et al., 2014). We have developed a novel perfusion rotating wall vessel bioreactor that enables control of metabolism and provides gentle mechanical stimulation, which result in enhanced tissue formation (Cha et al., 2022) (Cha et al., 2022). In the future we expect to adapt the 3D hydrogel system described within using the novel perfusion system to further enhance differentiation and tissue formation. In the present study, lung ECM proteins, such as LAMA5 and FN1, were highly upregulated in the dynamic culture conditions across the culture time points. These two ECM proteins contribute to lung branching morphogenesis, vasculogenesis, and alveolarization. FN1 plays a role in key lung developmental processes, such as proliferation and differentiation (Roman, 1997). Hornberger et al. (2000) showed that the proliferation of human fetal ventricular myocytes depends on their ability to secrete collagen and fibronectin (Lelkes et al., 1998; Hornberger et al., 2000). In agreement with Hornberger et al. (2000), enhanced ECM production in 3D dynamic conditions correlated with higher proliferation and faster differentiation on day 20 Thus, 3D culture has many advantages that support and enhance endoderm induction. These include flow integration, supplying oxygen and other essential nutrients to the appropriate sites, interaction between different cell types, a higher degree of structural complexity, and maintenance of homeostasis for longer than conventional methods (Bonaventure et al., 1994; Chang and Hughes-Fulford, 2009).

In summary, this study presents a novel approach for promoting the differentiation of human IPSCs towards type II pneumocytes in 3D culture using alginate encapsulation and a HARV bioreactor. The results suggest that dynamic culture provides an improved environment for cell growth and proliferation, and enhanced ECM production correlates with higher proliferation and faster differentiation. The developed method may have potential clinical applications to produce cells to be used in transplants. However, further investigation is needed to confirm the expression of mature surfactant proteins and lipid profile in human IPSC-derived type II pneumocytes-like cells. Moreover, evaluating the protocol on other cell lines will assess its reproducibility, competence, and therapeutic readiness in animal studies and in future clinical trials.

## Data Availability

The original contributions presented in the study are included in the article/Supplementary Material further inquiries can be directed to the corresponding author.
