# Supplementary material for: Differentiation of human induced pluripotent stem cells into functional lung alveolar epithelial cells in 3D dynamic culture
Source: Front Bioeng Biotechnol. 2023 Jun 14;11:1173149. doi: 10.3389/fbioe.2023.1173149 (PMC10303808; doi:10.3389/fbioe.2023.1173149)
Supplement: Supplementary file 1 [file DataSheet1.docx]

Supplementary Material


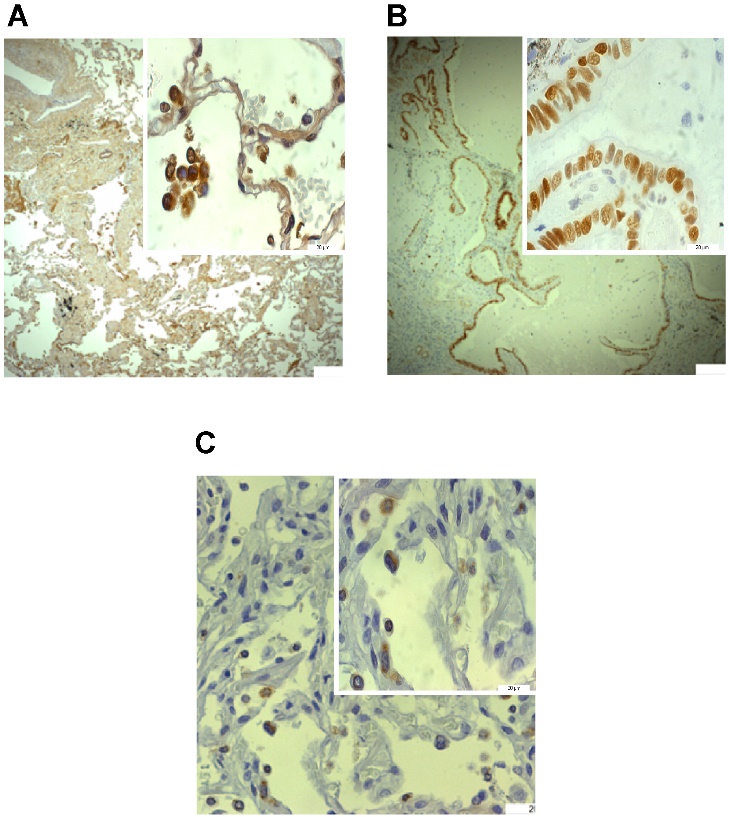


Supplementary Figure 1. Positive tissue controls used for immunohistochemistry. **(A)** Sections of normal human lung stained with anti-SFTPB polyclonal antibody (AB3786-Millpore, 1:4000-1:8000 dilution). **(B)** Sections of human lung adenocarcinoma stained with anti- ANTI-TTF rabbit monoclonal IgG (AB76013-Abcam, 1:100-1:1000 dilution). **(C)** Sections of normal human lung stained with anti-SFTPC rabbit polyclonal antibody (AB3786-Millpore, 1:1000-1:2000 dilution). Scale bars, 500 µm and 20 µm. Tissues were provided by the National Heart & Lung Institute Faculty of Medicine, Imperial College London. TTF, thyroid transcription factor 1.


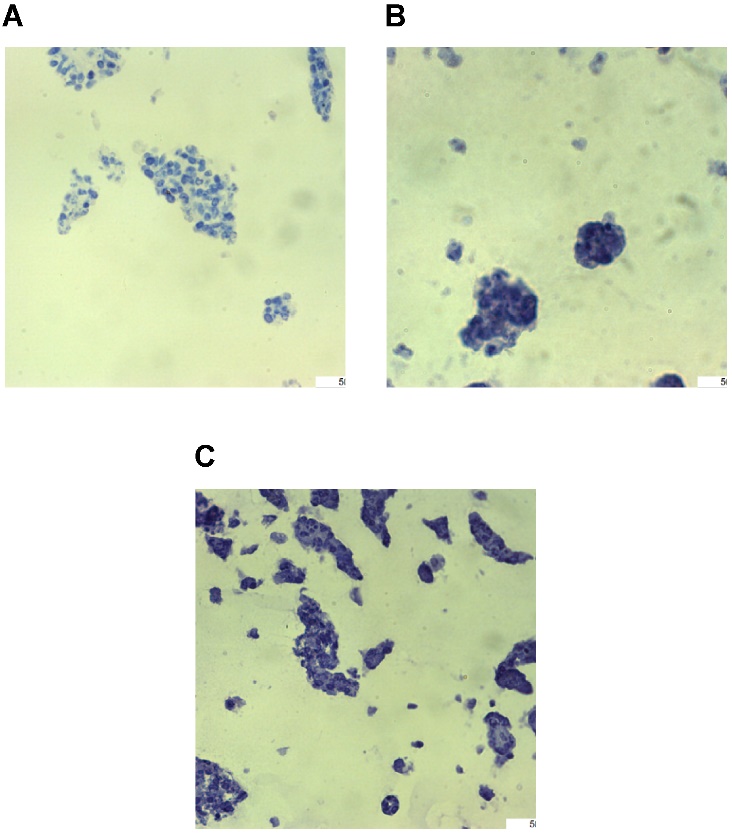


Supplementary Figure 2. Negative tissue controls used for immunohistochemistry. Immunohistochemical analysis of sections of alginate beads maintained in mTeSR™1. **(A)** ANTI-TTF (AB76013-Abcam, 1:100-1:1000 dilution) . **(B)** SFTPB (AB3786-Millpore, 1:4000-1:8000 dilution) . C) Immunohistochemical analysis of SFTPC using anti-PROSP-C Polyclonal antibody Rabbit (AB3786-Millpore, 1:1000-1:2000 dilution) . Scale bars, 50 µm. TTF, thyroid transcription factor 1.


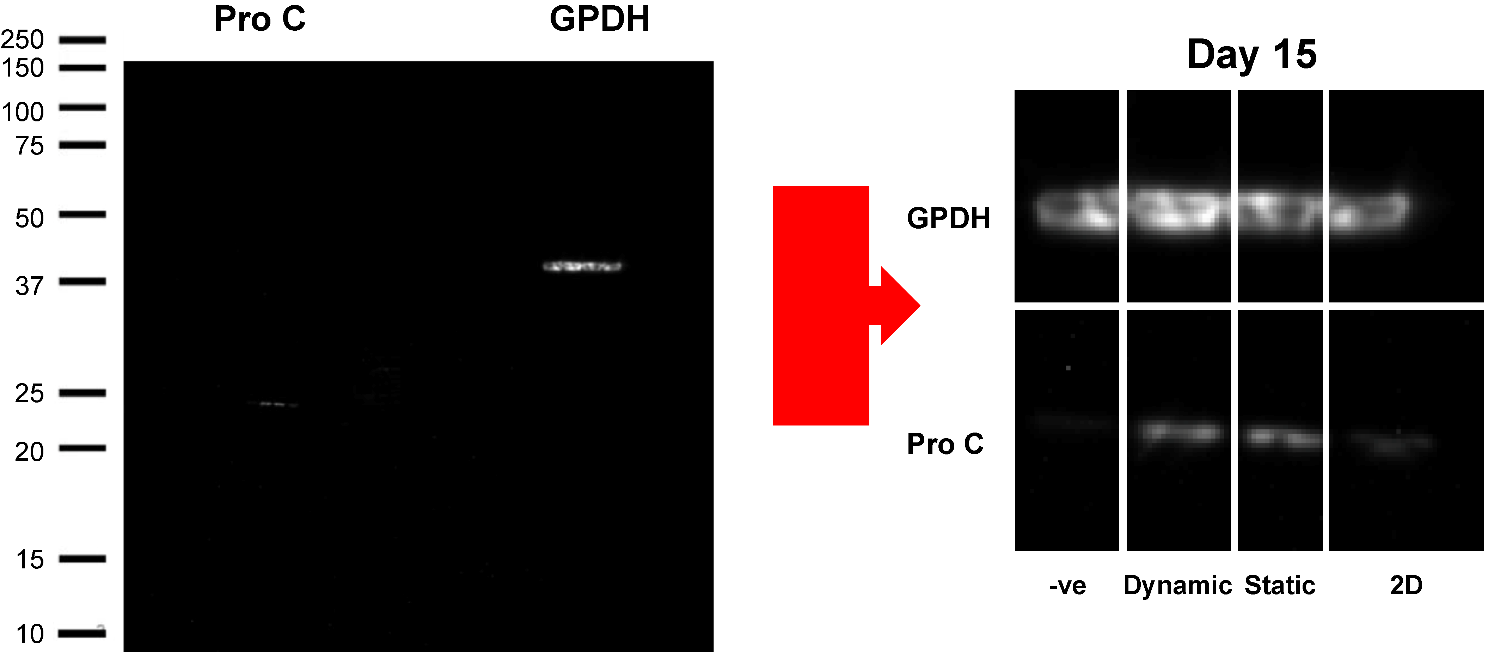


Supplementary Figure 3. Western blot showing pro-SFTPC on day 15 for 3D dynamic, static, and 2D cultures. 2D, two dimensional; pro-SFTPC, surfactant protein C.
